# Euthanasia

**Published:** 1885-11-20

**Authors:** 


EUTHANASIA.—By euthanasia is meant an easy death. The term has, of late, been applied to methods for the painless extinction of life in the lower animals. Carbonic oxide, chloroform, carbon bisulphide, and common coal gas are the agents used for the purpose.

